# Integrated Care in Atrial Fibrillation: A Multidisciplinary Approach to Improve Clinical Outcomes and Quality of Life

**DOI:** 10.3390/healthcare13030325

**Published:** 2025-02-05

**Authors:** Ana Mónica Machado, Fernanda Leite, M. Graça Pereira

**Affiliations:** 1 Research Centre in Psychology, School of Psychology, University of Minho, 4720-057 Braga, Portugal; anamonicamachado2021@gmail.com; 2 Department of Transfusion Medicine, Santo António University Hospital Center, 4040-342 Porto, Portugal; fernandajtleite@hotmail.com; 3 i3S-Institute for Health Research and Innovation, University of Porto, 4200-135 Porto, Portugal; 4 Public Health and Forensic Sciences, and Medical Education Department, Faculty of Medicine, University of Porto, 4099-002 Porto, Portugal

**Keywords:** atrial fibrillation, integrated care, quality of life, multidisciplinary

## Abstract

**Background:** Atrial fibrillation (AF) is the most common arrhythmia globally, associated with serious complications such as stroke and heart failure, as well as significant impacts on patients’ quality of life. **Objectives**: This theoretical article explores the role of integrated care in the management of AF, highlighting the need for a multidisciplinary approach that goes beyond rhythm and heart rate control. **Methods**: Through a review of the literature, this article explores the prevalence of AF, the challenges of diagnosis, the socioeconomic and psychological impact, as well as the benefits of integrating medical, psychological, and social interventions, drawing on insights from studies about integrative care in AF. **Results**: The findings highlight the challenges of managing AF, including its high prevalence, complex diagnosis, and significant socioeconomic and psychological impacts on patients. Integrated care models, combining medical, psychological, and social interventions, improve treatment adherence, reduce complications like stroke and heart failure, and enhance patient quality of life. **Conclusions**: Integrated care models hold significant promise in improving outcomes in AF patients through structured, multidisciplinary approaches. Evidence supports reductions in cardiovascular events, hospitalizations, and mortality when adhering to clinical guidelines, emphasizing patient education, and implementing individualized care strategies. Despite challenges, like regional disparities and suboptimal implementation, the integration of multidisciplinary teams and emerging technologies offers a way to enhance care delivery and accessibility. Future efforts should focus on personalizing care, promoting professional collaboration, and taking advantage of technological advances to optimize AF management and promote sustainable health systems.

## 1. Introduction

AF is the most prevalent sustained cardiac arrhythmia in clinical practice [1,2,3], representing a significant global cardiovascular health issue challenge due to its rising prevalence, especially in aging populations [3,4]. Recent data from the Global Burden of Disease (GBD) study underscores the significant global increase in atrial fibrillation (AF) prevalence over the past three decades [5]. Cheng et al. (2024) [5] reported that the number of individuals living with AF/atrial flutter surged from 33.5 million in 1990 to over 59 million by 2021, driven primarily by the aging populations and the rising prevalence of modifiable risk factors such as hypertension, obesity, and diabetes mellitus. Similarly, Dong et al. (2023) [6] highlighted that the socioeconomic disparities and lifestyle factors, including physical inactivity, unhealthy diets, and excessive alcohol consumption, have significantly influenced this trend. However, the true prevalence of AF is underestimated, as early diagnosis remains a critical challenge, as many patients are only identified when symptoms develop, or a stroke occurs [7].

AF is a significant contributor to high rates of cardiovascular morbidity and mortality [2,3,8], being independently associated with an increased risk of adverse cardiovascular events, including heart failure and stroke [2,3], as well as an elevated risk of cognitive decline and dementia, even in the absence of a stroke history [2,3]. Although advancements in treatment have improved life expectancy for AF patients, they still experience, on average, a two-year reduction in lifespan compared to those without the condition [9]. Moreover, indirect mortality due to complications such as stroke and heart failure remains a critical concern [9,10].

There are several risk factors linked with the development of AF, but age plays a predominant role due to the structural and functional cardiac changes associated with aging [11]. Furthermore, the chance of developing AF increases by factors such as hypertension, heart failure, diabetes mellitus, chronic kidney disease, obesity, obstructive sleep apnea, alcohol consumption, and smoking [2,3,11]. To effectively manage AF, a comprehensive approach is essential [2,3], which includes addressing acute treatment and underlying cardiovascular conditions, optimizing anticoagulation therapy, improving rate control, managing rhythm, offering psychological and social support, and integrating ablation strategies when appropriate [2,3,12]. Evidence from trials such as CABANA [13] and CASTLE-AF [14] highlights the role of ablation in reducing symptom burden, improving quality of life (QoL), and, in selected cases, lowering heart failure hospitalizations and cardiovascular mortality. Incorporating ablation strategies, at the correct time within an integrated care model, ensures that AF management is both comprehensive and personalized regarding the patients’ diverse needs [2,13]. Additionally, the risk of complications, such as stroke and heart failure, requires continuous monitoring and coordinated care, making the management of AF a multifaceted and resource-intensive process [2,3].

Beyond these clinical aspects, AF significantly impacts patients’ QoL, particularly in physical, emotional, and social domains [2,3,15,16,17], and impacts healthcare systems all through Europe [2,3,16]. Reduced functional capacity, social isolation, and emotional distress are frequently caused by symptoms, including fatigue, palpitations, dizziness, and uncertainty [2,3,15,17,18]. The literature shows that, given its complexity, AF patients have a lower QoL than the general population and those suffering from other cardiovascular pathologies [2,3,19].

Managing AF involves substantial clinical challenges, and the complexity of the risk factors and associated comorbidities makes the diagnosis and treatment of AF a multidisciplinary task [2,3,18]. Evidence of the challenges can be found in the ongoing difficulties associated with treatment adherence, which poses a significant risk to achieving long-term outcomes.

Addressing these factors is essential to improving outcomes and providing comprehensive treatment for people with AF.

## 2. From Guidelines to Practice: Integrated Care in AF

The increasing prevalence of chronic diseases [20], along with advances in medical treatments and technology [21], has resulted in more complex care and treatment pathways [22,23]. This advance calls for a reorientation in healthcare delivery toward patient-centered models that meet the multiple needs of individuals suffering from chronic diseases [24].

Integrated care is a multifaceted approach aimed at coordinating and harmonizing fragmented health systems to provide continuous, patient-centered care [25,26]. It seeks to address the challenges of aging populations, chronic comorbidities, and increasing healthcare demands by optimizing physical, mental, and social care across organizational boundaries [25,27]. While this concept is widely accepted, its implementation remains complex due to structural and cultural differences between services, financial incentives, and outdated role expectations [27]. Integrated care can improve patient experience, reduce duplication, and promote holistic care [27]. However, its success ultimately depends on healthcare workers’ skills, behavior, and engagement [27]. Integrated care should be seen not as a singular intervention, but as a holistic framework that promotes interdisciplinary collaboration to improve patient outcomes, enhance service efficiency, and enrich healthcare experiences [2,3].

In the AF context, integrated care is increasingly recognized as the optimal approach to managing AF [2,3], particularly given its chronic and complex nature. The effective management of AF is essential to mitigate its negative impact on patients’ health and QoL [2,3,28]. AF management involves a comprehensive approach encompassing continuous cardiac monitoring, therapeutic strategies to control heart rate or rhythm, and the prevention of complications, particularly stroke QoL [2,3,28]. Anticoagulation remains essential in AF management due to its efficacy in reducing thromboembolic events [29,30]. While vitamin K antagonists (VKAs) like warfarin effectively lower stroke risk, their narrow therapeutic window, frequent INR monitoring requirements, and bleeding risks, particularly intracranial hemorrhage, pose significant challenges [31]. Direct oral anticoagulants (DOACs) have largely supplanted VKAs as the preferred anticoagulation therapy [32]. With predictable pharmacokinetics and no need for routine monitoring, DOACs have demonstrated comparable or superior efficacy and safety in preventing stroke and systemic embolism [32,33,34]. However, the bleeding risk associated with anticoagulation necessitates careful risk assessment using tools like the HAS-BLED score and individualized management strategies, including dose adjustments and close monitoring for high-risk patients [2,3].

Restoring and maintaining sinus rhythm is another critical goal in AF management, especially for symptomatic patients [2,3]. Sinus rhythm restoration alleviates symptoms such as palpitations, fatigue, and dyspnea while improving exercise tolerance [2,3]. Electrical cardioversion is a highly effective method, achieving high success rates in recent-onset or persistent AF, often combined with anticoagulation to mitigate thromboembolic risks [2,29]. Pharmacological cardioversion, using antiarrhythmic drugs, offers a less invasive alternative [2,29]. Catheter ablation, the most effective procedure for preventing AF recurrence, is particularly recommended for symptomatic patients with paroxysmal or persistent AF refractory to antiarrhythmic drugs, demonstrating superior efficacy in maintaining sinus rhythm [35].

Managing comorbidities is essential for improving AF outcomes, as conditions such as hypertension, diabetes, obesity, sleep apnea, and heart failure often exacerbate disease progression [2,3,36]. The effective control of these comorbidities reduces the risks of stroke, heart failure, and arrhythmia recurrence while enhancing overall prognosis and QoL [2,3]. A multidisciplinary approach, incorporating comprehensive comorbidity management alongside rhythm and rate control, underscores the importance of tailored, patient-centered strategies in optimizing AF treatment [2,3].

The European Society of Cardiology (ESC) [2,3] guidelines advocate an integrated care model that combines patient-centered management with a multidisciplinary approach. Although significant progress has been made in treatment and in elucidating the pathophysiological mechanisms that contribute to AF [11,28], there is a need for innovative methods to enhance the treatment of AF and its related conditions [22,23].

The integrated patient-centered model emphasizes a multidisciplinary team and comprehensive management, including AF treatment, thromboembolic prevention, and control of comorbid conditions and cardiovascular risk factors AF [2,3]. Despite these advancements and international guidelines advocating for integrated care, its implementation remains inconsistent [22,23]. Further research is essential to refine d therapy for AF, optimize rhythm control strategies, enhance anticoagulation safety, and promote early, effective interventions to mitigate complications [22,23]. Such integrated care is crucial in ensuring consistent, accessible, and comprehensive management for all patients with AF, contributing significantly to improved clinical outcomes and enhanced QoL.

The proper management of AF is crucial to minimize its negative impact on patients’ health and QoL [2,3,37]. The effective management of AF requires a multidimensional strategy that addresses acute care, the treatment of underlying cardiovascular conditions, stroke prevention, rate control, and rhythm control [2,3]. Recent international clinical guidelines emphasize an integrated care approach to improve both the physical and psychosocial well-being of patients, while increasing healthcare efficiency [2,3,8]. The ESC [2,3] and the Canadian Cardiovascular Society (CCS) [38] underscore the importance of integrated care in AF management, and published guidelines to adopt. These guidelines focus on four essential pillars: active patient participation, collaboration among multidisciplinary teams, use of technological tools, and access to all treatment alternatives [2,3,8]. Based on these four essential pillars, each plays a crucial role in optimizing integrated care delivery for AF patients, ensuring the comprehensive and effective management of the condition [2,3,8]. Patient involvement is essential in the healthcare process and requires the implementation of a patient-centered approach [2,3,8]. In addition to receiving adequate education, patients must be included in the decision-making process regarding their treatment [2,3,8]. In addition, they should be empowered to take an active role in managing their health (i.e., adherence to prescribed treatment plans, addressing their risk factors, and adopting a healthy lifestyle) [2,3,8]. Also, a multidisciplinary team approach is recommended due to the complex nature of AF management, rather than relying on a single healthcare provider [2,3,8]. t The team approach requires effective collaboration and communication between members of the treatment team, along with coordinated efforts to avoid any fragmentation in patient care [2,3,8]. The integration of mobile health and e-health technologies into healthcare is gaining prominence [21], thus facilitating the self-management of their health problems. These technologies aim to improve integrated care, contributing to the provision of complete and personalized diagnostic and therapeutic approaches [21]. However, as highlighted by the DIGI-COVID study [39], the effectiveness of e-health tools is influenced by digital health literacy, particularly among elderly patients—a population with a high prevalence of atrial fibrillation (AF). The DIGI-COVID study underscores that frail and older cardiology patients often face significant challenges in using digital technologies due to limited digital literacy [39]. This gap creates a discrepancy between the potential benefits of mHealth tools and their actual implementation in this vulnerable population [39]. To bridge this gap, tailored educational programs, simplified user interfaces, and caregiver involvement are essential to ensure equitable access and effective utilization of digital health solutions in elderly AF patients [39]. Without addressing these barriers, the promise of digital tools in improving AF care may remain unfulfilled for this critical population segment. Finally, ensuring that patients have access to a full spectrum of AF management options—including rhythm control strategies, anticoagulation therapies, and risk factor modification—is a critical component of integrated care [2,3].

The integrated care model delivery standardizes care and is associated with better patient outcomes and a lower occurrence of complications such as stroke and heart failure while emphasizing patient-centered, multidisciplinary approaches [2,3,8,40]. Involving a wide range of healthcare professionals ensures the continuity of care and supports adherence to evidence-based treatments [2,3,8,40,41]. This collaborative framework recognizes that AF management goes far beyond controlling heart rate and rhythm and involves lifestyle modification, patient education, and psychological support [2,3,8,40,41].

The ESC’s ABC pathway (avoid stroke, better symptom management, cardiovascular risk reduction), as outlined in the ESC guidelines, is one intervention that provides a systematic framework for achieving key goals in AF management: preventing strokes, managing symptoms effectively, and ensuring thorough cardiovascular risk management [2,3,40]. This is a comprehensive approach to AF treatment that aligns with the essential pillars of integrated care, emphasizing a comprehensive and patient-centered approach to AF management [2,3,42].

However, the implementation, effectiveness, and role of these integrated care models in AF remain unclear, and there seems to exist a gap between practice and theory [43]. So, how can we better understand the true impact of these integrated care models on AF management?

### Insights from Research

The traditional medical model proves highly effective in delivering acute care but falls short in managing chronic conditions [44]. With the growing burden of chronic cardiovascular diseases [45], the economic sustainability of this model is increasingly in question [28]. Furthermore, chronic illnesses require redesigned care systems to manage the disease process effectively and support patients over time [46].

In the context of AF, several studies have highlighted the fact that integrated models are associated with a reduction in all-cause mortality [22,28] and fewer cardiovascular events [28,42,47]. A recent systematic review and meta-analysis [48] showed an improvement in the outcomes of AF patients with integrated care, showing also an association between integrated care and a reduction in the risk of all-cause mortality.

Another systematic review and meta-analysis [47] showed that integrated care can reduce the risk of all-cause mortality, cardiovascular mortality, and cardiovascular hospitalizations in patients with AF compared with usual care.

Several randomized controlled trials (RCTs) have explored the effectiveness of integrated care models in managing AF, demonstrating their impact on improving clinical outcomes, reducing adverse events, and enhancing patient adherence to treatment guidelines.

The research from Inglis et al. (2004) [49] explored the impact of a nurse-led, multidisciplinary, home-based intervention on patients with chronic AF. The study involved two RCT(s) conducted in, South Australia. A total of 762 patients with various chronic disease states were initially studied. From this cohort, 152 patients (16% of the total) with chronic AF were identified and included in the analysis. Patients were recruited during acute hospitalization and subsequently discharged home. Participants were randomly allocated to either the home-based intervention group or the usual post-discharge care group. The intervention group received comprehensive management involving a qualified cardiac nurse who performed home visits. The intervention aimed to establish a comprehensive clinical and sociodemographic patient’s profile, optimize access to multidisciplinary management, including collaboration with primary care physicians, specialists, community pharmacists, and other allied health professionals, ensure the quality use of medicines, patient education, and the early detection of clinical deterioration, and facilitate patient-initiated contact for concerns. The study monitored all inpatient and outpatient hospital activities through a computerized medical records system. Results showed that the intervention was associated with better health outcomes in both patients with chronic AF and those with concurrent heart failure. Specifically, the intervention led to a reduction in recurrent hospitalizations and mortality rates compared to usual post-discharge care. The findings suggested that the benefits of this intervention could extend beyond immediate post-discharge care, highlighting the potential for long-term improvements in patient management and outcomes for those with chronic AF [49].

The study by Hendriks et al. (2012) [50], a prospective RCT, was conducted at the Maastricht University Medical Centre, recruiting patients from January 2007 to December 2008, with a follow-up period of at least one year. Patients aged 18 years or older were randomly assigned to either nurse-led care or usual care. The nurse-led care group implemented a care model managed by nurse specialists using decision-support software aligned with clinical guidelines under cardiologist supervision. Initial assessments included laboratory tests, electrocardiograms, Holter monitoring, and echocardiography, ensuring a comprehensive evaluation before initiating care [50]. This rigorous design facilitated a detailed comparison between integrated, nurse-led care and routine care, emphasizing a multidisciplinary, guideline-based approach to AF management [50]. Results showed that nurse-led care was associated with a significantly lower occurrence of cardiovascular hospitalization and cardiovascular death [50]. Specifically, there was a 35% relative risk reduction in adverse events for the nurse-led care group. Also, the study found that cardiovascular death occurred in 1.1% of the nurse-led care group versus 3.9% in the standard care group, and the rates of cardiovascular hospitalization were 13.5% in the nurse-led care group compared to 19.1% in the usual care group [50]. Additionally, patients in the nurse-led care group demonstrated significantly better adherence to clinical guidelines compared to those receiving standard care [50]. This was attributed to the structured approach of the nurse-led model, which used decision-support software to enhance guideline implementation [50]. Overall, the trial showed that nurse-led integrated care for ambulatory patients with AF significantly reduced cardiovascular hospitalizations and mortality compared to standard care, while also improving adherence to treatment guidelines [40]. The findings highlight the effectiveness of integrated, nurse-led care as a superior alternative for managing chronic AF [50].

Although this trial showed that nurse-led care was superior to standard care, the study by Wijtvliet et al. (2020) [51] failed to show that nurse-led care was superior to usual care. The RACE 4 trial (Rate Control vs. Electrical Cardioversion Trial 4) was a multicenter, randomized study designed to compare nurse-led care with standard care provided by cardiologists for patients with AF. A total of 1375 patients with AF were randomized into two groups: 671 patients received nurse-led care, and 683 patients were managed with usual care [51]. The nurse-led care model involved specialized nurses using decision-support tools in collaboration with cardiologists to improve adherence to clinical guidelines and promote coordinated care delivery [51]. The study evaluated guideline adherence, treatment strategies, and patient outcomes, including hospitalizations for arrhythmia and heart failure [51]. The study also analyzed the use of diagnostic procedures and the effectiveness of therapeutic interventions. The results showed that nurse-led care did not significantly reduce the risk of cardiovascular death or hospital admissions compared to usual care provided by cardiologists [51]. Cardiovascular death and hospital admissions showed similar event rates between the two groups, and groups had comparable rates of appropriate anticoagulation treatment, with 81% in the nurse-led care group and 82% in the usual standard care group receiving anticoagulation following guidelines [51]. The effectiveness of nurse-led care varied significantly based on the experience of the centers involved. In experienced centers, nurse-led care showed a potential benefit, while in less experienced centers, the opposite was true [51]. However, the study noted a lack of effect of nurse-led care on patient knowledge and QoL, suggesting that while nurse-led care is safe, it may not enhance these aspects of patient care [51].

Another study exploring a new management strategy for patients with chronic AF is the study by Stewart et al. (2015) [52]. This pragmatic, multicenter RCT, known as SAFETY, investigates whether a disease-specific approach can reduce hospital readmissions and increase survival rates [52]. With a total of 335 participants, the study compares standard management practices with innovative SAFETY intervention, which includes personalized follow-up care and support. Participants allocated to the SAFETY intervention received enhanced clinical surveillance and support that included home visits by a cardiac nurse with postgraduate training within 7–14 days post-discharge, the use of the GARDIAN method to assess individual risk and needs, and comprehensive reporting to the medical team outlining key findings and recommendations for optimizing treatment. Participants were followed for a minimum of 24 months, with prescheduled health reviews at 12 and 24 months, including ECG Holter monitoring. This strategy significantly reduced recurrent hospital admissions and improved survival compared to standard care, suggesting potential advantages of a disease-specific management approach for chronic AF patients [52].

Carter et al. (2016) [53] showed that an integrated management approach to AF improved patient outcomes, reducing AF-related hospitalizations and enhancing symptom management and QoL. The research used a “before-and-after” study design, delivering a nurse-led intervention. Patients presenting with new-onset AF to the emergency department were studied in two phases: the usual-care phase (before) and the AF clinic phase (after). The study included patients aged 18 years and older who were diagnosed with new-onset AF confirmed by electrocardiography between 1 January 2009 and 31 January 2014. The study showed a reduction in adverse outcomes for the AF clinic group as well as improved guideline adherence [53]. By emphasizing multidisciplinary collaboration, patient education, and guideline adherence, the study highlighted the effectiveness of integrated care in reducing the healthcare burden of AF [53].

The integration of AF management into primary care settings has also shown promising results [54]. A study, conducted in the Netherlands, explores the effectiveness and safety of implementing integrated care for AF within primary care settings [54]. The ALL-IN trial showed that implementing integrated care in primary care settings resulted in a 45% reduction in all-cause mortality compared to usual care [54]. The ALL-IN trial was a cluster-randomized, pragmatic, non-inferiority trial. This design was chosen to compare integrated care in primary care settings with the usual care provided by cardiologists and anticoagulation clinics. The intervention involved the structured management of AF by general health professionals and practice nurses, focusing on integrated care. The control group received usual care, which typically involved consultations with cardiologists or AF nurses [54]. The results showed a significant reduction in all-cause mortality among elderly AF patients receiving integrated care in primary care settings; specifically, there was a 45% reduction in all-cause mortality in the intervention group compared to the usual care group [54]. Also, the results indicated a 45% reduction in the risk of death with integrated care. The trial also found a reduction in non-cardiovascular mortality for patients in the intervention group. The integrated care approach included regular check-ups by trained nurses, the monitoring of anticoagulation therapy, and addressing comorbidities, which contributed to the observed improvements in patient outcomes. The findings of this study support the notion that AF management should not be viewed merely as a heart rhythm disorder but rather as part of a systemic condition that requires comprehensive care, particularly in elderly patients with multiple comorbidities [54].

The incorporation of mobile health technology into integrated care strategies has further advanced AF management [55,56]. A cluster RCT using an AF mobile app [57] to deliver AF care showed a reduction in readmission rates and adverse events, suggesting that technology-supported integrated care can improve patient outcomes. The mAFA II trial was a prospective, multicenter, cluster-randomized controlled trial. It aimed to evaluate the effectiveness of a mobile health (mHealth) technology-supported integrated management strategy for patients with AF compared to usual care [57]. This intervention involved the use of a user-friendly mobile application for both doctors and patients. This platform provided clinical decision-support tools, educational materials, and strategies for patient involvement and self-care, all aimed at facilitating guideline-based treatment recommendations [57]. Patients were followed up in outpatient clinics at 6 and 12 months to assess clinical events and outcomes related to AF management. The study concluded that an integrated care approach to AF management, supported by mHealth technology, effectively reduces the risks of rehospitalization and clinical adverse events, highlighting the potential of mobile health solutions in improving patient outcomes in AF management [57].

Luo and colleagues (2022) [55] conducted a study to evaluate the cost-effectiveness of implementing mHealth-based integrated care for AF through a model-based health economic assessment. The study, conducted in China, compared outcomes of mHealth-based care and usual care in a hypothetical cohort of patients with AF. The results indicated that mHealth applications could potentially serve as a cost-effective means of enhancing and coordinating care within the ABC pathway [55].

The study by Fuenzalida et al. (2017) [58] found that education provided by emergency care nurses at discharge improved patient knowledge, reduced hospital readmissions, and enhanced self-management in AF patients, leading to better long-term outcomes. This study was designed as a prospective, randomized, controlled intervention conducted in the emergency department of a tertiary hospital in Barcelona, Spain. It included patients aged 18 years and older who were diagnosed with AF based on electrocardiography and were being discharged at the time of inclusion. Patients were included only once while the study was ongoing, regardless of the number of emergency visits. So, patients were randomized into an intervention group that received additional educational support and a control group that received standard care. The intervention group received training from a nurse who provided standardized information based on ESC guidelines. These guidelines included explanations of AF, potential complications, treatment precautions, warning signs, and a personalized leaflet about their medication. At one-year post-intervention, follow-up included a review of each patient’s electronic medical record to assess complications attributable to AF or its treatment, the number of visits to the emergency (both total and AF related), the number of hospital admissions (both total and AF related), and mortality rates. Results showed that treatment-related complications and death were significantly lower in the intervention group compared to the control group, and mortality was lower in the intervention group, with 22.4% of patients in this group dying compared to 34.7% in the control group, although this difference was not statistically significant [58]. The results also indicated that the educational intervention provided by emergency nurses at discharge had a positive impact on patient prognosis, leading to fewer complications associated with AF or its treatment over the long term [46]. These outcomes underscore the importance of health education in improving outcomes for patients with AF [58].

The study addressing the multifaceted intervention to improve treatment with oral anticoagulants in atrial fibrillation (IMPACT-AF) by Vinereanu et al. (2017) [59] aimed to enhance the use of oral anticoagulants in patients with AF through a comprehensive educational approach. By comparing a quality improvement intervention to usual care, the researchers sought to determine the effectiveness of their methods in increasing anticoagulant treatment over a year [59]. Clusters in five participating countries were randomized to receive either a quality improvement educational intervention (intervention group) or standard care (control group). The intervention included two key educational components to improve knowledge and communication about oral anticoagulation therapy. For patients and families, it provided educational brochures, web-based resources, and videos designed to enhance their understanding of the therapy. Patients were also encouraged to have discussions with healthcare providers to explore the benefits and risks of oral anticoagulation, promoting shared decision-making. For healthcare providers, the intervention involved systematic reviews of current guidelines and regular emails containing relevant articles, webinars, podcasts, and specialized monographs aimed at keeping healthcare professionals informed about the latest evidence and best practices in anticoagulation management. This dual-target strategy was designed to improve patient engagement and provider adherence to evidence-based practices [59]. The trial showed a significant increase in the use of oral anticoagulants among patients in the intervention group and also observed a reduction in the incidence of strokes among patients in the intervention group compared to the control group [59]. Additionally, the positive effects of the educational intervention were consistent across various countries and key patient subgroups, indicating that the intervention was effective in diverse settings [59]. Overall, the IMPACT-AF trial provided strong evidence that educational interventions can effectively enhance the use of oral anticoagulants in patients with AF, leading to improved patient outcomes, which has important public health implications [59].

Another study aiming to explore how tailored education could enhance patients’ QoL, reduce symptom severity, and potentially lower the rate of rehospitalization, with a focus on patient-centered care, is the study by Bowyer et al. (2017) [60]. The study employed a randomized controlled trial design to evaluate the effects of a nurse-led educational intervention on patients undergoing catheter ablation for AF. Patients were randomized into one of two groups before undergoing the ablation procedure. They were allocated to either the nurse intervention group, which received additional structured educational support, or the control group, which received standard physician care only. In the nurse intervention group, participants received structured education from a nurse specialized in arrhythmia management. Educational sessions were delivered at five specific time points: two face-to-face sessions, one at admission, and another before discharge (30 min each), and three follow-up telephone sessions at two weeks, one month, and three months post-procedure (each lasting 5–10 min). The educational content provided during these sessions included an overview of how the heart functions, the causes and risk factors associated with AF, AF symptoms, the goals of treatment, a detailed review of the ablation procedure, and recommended lifestyle modifications to support heart health. This intervention was designed to empower patients with knowledge and improve their ability to manage their condition effectively [60]. The nurse intervention group showed significant improvements in QoL as well as improvements in AF symptoms [60]. The study also suggested that the educational intervention might have positively influenced the patients’ interpretation of their symptoms and reduced anxiety, which could have contributed to the observed improvements in symptom frequency and QoL [60].

The ABC pathway was found to be linked to a reduced likelihood of negative health outcomes [3]. Several studies have shown that the ABC pathway effectively reduced the risk of adverse outcomes in patients with AF, e.g., Refs. [23,61,62,63].

In 2019, Pastori et al. [64] assessed the integrated care management of AF patients using the ABC pathway within the ATHERO-AF study cohort. The results of this prospective single-center cohort, which included 907 consecutive patients with nonvalvular AF on vitamin K antagonists, showed that the ABC pathway significantly reduced the risk of cardiovascular events in AF patients [64]. Specifically, patients who followed the ABC pathway experienced substantially lower rates of cardiovascular events, highlighting the benefits of integrated care models in managing AF and reducing cardiovascular risks [64].

Yoon et al. (2019) [65] conducted a nationwide cohort study to assess the effect of compliance with the simple ABC pathway on clinical outcomes in AF patients. Using data from the Korea National Health Insurance Service, the study included 20,4842 patients with nonvalvular AF who were enrolled from 1 January 2005 to 31 December 2015. The study found that adherence to the ABC pathway led to significantly improved clinical outcomes, including lower rates of all-cause mortality [65]. The pathway was shown to enhance overall AF management [65]. These findings emphasize the effectiveness of the ABC pathway in improving patient outcomes on a population level, supporting its use in integrated care strategies for AF [65].

Analyzing data from the AFFIRM trial (A Comparison of Rate Control and Rhythm Control in Patients with Atrial Fibrillation, 2002), the study by Proietti and colleagues (2018) [61] aimed to demonstrate how the ABC pathway can lead to better patient care and outcomes. Patients were categorized into the ABC group who fulfilled all three criteria of the ABC pathway—and into the non-ABC group—who fulfilled fewer than three criteria. The study showed that implementing the integrated care approach based on the ABC pathway significantly improved patient outcomes when compared to non-ABC management [61]. The results showed that patients in the ABC group had a markedly reduced risk of clinically relevant outcomes, including all-cause mortality, stroke, major hemorrhage, and cardiovascular death, as well as first hospitalization [50]. In addition, the ABC pathway led to lower hospitalization rates, including a reduced risk of multiple hospitalizations, a lower total number of admissions, and fewer cumulative days spent in the hospital [61]. It was also observed that greater adherence to the integrated care components of the ABC pathway corresponded to additional reductions in clinically relevant outcomes [61]. Thus, the results highlight the ABC pathway as a comprehensive approach to AF management, effectively addressing symptom control, hospitalization burden, and overall cardiovascular health [61].

Proietti et al. (2020) [66] investigated the importance of integrated care approaches for managing AF, particularly in patients with additional health conditions. The analysis utilized pooled data from the ‘Stroke Prevention using an Oral Thrombin Inhibitor in Patients with Atrial Fibrillation’ (SPORTIF) III and V trials [67]. These trials compared a direct thrombin inhibitor (ximelagatran), to warfarin, in patients with nonvalvular AF. Only patients assigned to the warfarin arms of the study were included in the analysis to ensure a representative cohort. The authors focused on three major metabolic comorbidities: diabetes mellitus, chronic kidney disease, and metabolic syndrome. The study found that integrated care, through adherence to the ABC pathway, was associated with a significant reduction in the risk of adverse cardiovascular events and all-cause mortality among high-risk AF patients with metabolic comorbidities such as diabetes mellitus, chronic kidney disease, and metabolic syndrome [66]. The analysis highlighted that patients with specific metabolic comorbidities are at a particularly higher risk for major adverse outcomes [66]. The ABC pathway-compliant management was inversely associated with the risk of composite outcomes in these subgroups, indicating that tailored management strategies can improve outcomes in high-risk populations [66].

Additional evidence comes from Proietti et al. (2021) [62], whose study, conducted by a team of experts across Europe analyzed data from the ESC-EHRA EORP Atrial Fibrillation General Long-Term Registry, that highlighted how adherence to the ABC pathway could improve outcomes for patients with AF. The study found that clinical management adherence to the ABC pathway was significantly associated with a lower risk of cardiovascular events, cardiovascular death, and all-cause death in AF patients [62]. The study emphasized the importance of an integrated care approach in managing AF, highlighting that a single disease management strategy could not be sufficient due to the complexity of comorbidities often present in AF patients [62]. The ABC pathway aimed to streamline care by focusing on anticoagulation, symptom management, and comorbidity management [62]. Although the findings are promising, the authors noted that their data should be considered as a ‘proof of concept’ and emphasized the need for more prospective studies to validate the results and further explore the impact of the ABC pathway on patient outcomes [62].

Focused on the holistic management of AF to enhance patient care and outcomes in the Balkan region, Kozieł et al. (2020) [68] explored how adherence to the ABC pathway could help manage symptoms and reduce stroke risk. The BALKAN-AF survey [68] was designed to collect real-world data on the management of patients with nonvalvular AF across various healthcare settings, including hospitals and outpatient clinics. The study revealed that adherence to the ABC pathway was suboptimal [68]. Particularly, less than half of AF patients received care, fully aligned with the ABC management strategy, underscoring a significant gap in guideline implementation [68]. Another notable result was the identification of nonadherence predictors. Patients aged 80 years or older and those with a history of bleeding were more likely to receive nonadherent management, highlighting potential challenges in treating older or high-risk populations [68]. On the contrary, several factors were independently associated with ABC adherence. These included being treated in a capital city, receiving care from a cardiologist, and the presence of comorbid conditions such as hypertension, diabetes mellitus, and multimorbidity [68]. These findings suggest that access to specialized care and the comprehensive management of coexisting conditions played crucial roles in improving adherence to guideline-based strategies [68]. These findings carry important clinical implications, particularly within the Balkan region, where significant gaps in adherence to the ABC pathway were observed [68]. This underscores the urgent need for targeted interventions and improved strategies to bridge the gap between evidence-based recommendations and real-world clinical practice. Finally, the study highlights the impact of adherence on patient outcomes, showing that adherence to the ABC pathway is associated with improved outcomes, including a reduction in cardiovascular events and lower healthcare costs. In contrast, nonadherence may contribute to suboptimal management and worse clinical outcomes. Therefore, the results underscore the challenges in implementing the ABC pathway in routine practice and emphasize the importance of addressing barriers to adherence [68]. Enhanced education, access to specialized care, and comprehensive management strategies are essential to optimize the treatment of atrial fibrillation in the Balkan region and beyond [68].

Other studies have shown similar trends, with Gumprecht et al. (2020) [69] reporting that adherence to the ABC pathway was linked to a reduction in all-cause mortality among AF patients in the Middle East. The research was conducted as an international cohort study based on a real-world observational registry of patients with AF from the Gulf SAFE registry [70]. This design allowed for the assessment of adherence to the ABC pathway and its outcomes in a diverse patient population. Out of the 2043 patients included in the Gulf SAFE registry, a total of 2021 patients were analyzed. The analysis revealed that adherence to the ABC pathway for integrated AF management was remarkably low, with only 168 patients (8.3%) receiving optimal care by the ABC pathway guidelines. This finding underscores a significant gap in the implementation of evidence-based integrated care within this population [69].

Another study that warrants attention is the work by Bucci et al. (2023) [23], which analyzed the outcomes associated with adherence to the ABC strategy among patients. With a diverse group of over 4000 participants, the findings aimed to enhance the quality of care for those living with AF [23]. The study was an observational analysis conducted within the Asia–Pacific Heart Rhythm Society (APHRS) Atrial Fibrillation registry, which enrolled inpatients and outpatients with atrial fibrillation (AF) from 2015 to 2017 across five Asian–Pacific countries (Hong Kong, South Korea, Japan, Singapore, and Taiwan). The study found that adherence to the ABC pathway was associated with improved clinical outcomes in patients [23]. Patients who followed the ABC criteria had a lower incidence of adverse outcomes compared to those who did not adhere [23]. Thromboembolic events, new or worsening heart failure, acute coronary syndrome, significant coronary artery disease requiring percutaneous coronary intervention, and all-cause death showed a significant reduction in risk for patients adhering to the ABC pathway [23]. Also, there was a notable decrease in all-cause mortality and cardiovascular death among patients who were adherent to the ABC pathway, indicating that integrated care management could lead to better survival rates [23]. Overall, the findings support the implementation of the ABC pathway as a standard approach in managing AF to enhance patient outcomes and reduce the risk of adverse events [23].

Similarly, Rivera-Caravaca et al. (2022) [63] explored how adherence to the ABC pathway could enhance the management of AF. The Murcia AF Project Phase II was a prospective cohort study aimed at evaluating the impact of adherence to the ABC pathway on clinical outcomes in patients with AF who were starting vitamin K antagonist therapy. Adherence to the ABC pathway was associated with significantly lower risks of adverse clinical outcomes in AF patients starting vitamin K antagonist therapy [63]. Specifically, patients who complied with the ABC pathway showed a reduced risk of all-cause mortality and composite outcomes such as ischemic stroke, major bleeding, and cardiovascular death [63]. The study showed that better adherence to the ABC pathway correlated with improved patient outcomes across several populations, including those with higher risk factors such as frailty, diabetes, and multiple comorbidities [63]. The application of the ABC pathway may, therefore, contribute to reduced healthcare costs related to cardiovascular events, highlighting its potential economic benefits alongside clinical improvements [63]. However, frailty is a critical factor influencing AF management in elderly patients, although often underappreciated. Frailty impacts clinical decisions, particularly regarding anticoagulation therapy, as it is a key driver of underdosing and poor drug adherence, which significantly impacts clinical outcomes [71]. Spruit et al. (2024) [71] highlighted that despite a decade of experience with direct oral anticoagulants (DOACs), frail elderly patients remain at higher risk for both thromboembolic and bleeding complications due to suboptimal anticoagulation. Underdosing, often driven by concerns about bleeding, compromises stroke prevention efficacy, while reduced adherence exacerbates the risk of adverse outcomes [71].

The EHRA expert consensus document [72] further underscores that frailty in the real world is associated with challenges in managing arrhythmias, including AF. Frail patients often face physiological vulnerabilities, multimorbidity, and reduced resilience, which complicate the implementation of guideline-recommended therapies [72,73]. These complexities need tailored strategies, such as multidisciplinary care models and patient-centered approaches, to optimize treatment adherence and outcomes. Given the findings, addressing frailty in AF management requires a comprehensive evaluation of the patient’s physical, cognitive, and social factors. Incorporating tools such as frailty indices or screening scales may id in identifying at-risk patients and guiding personalized therapeutic strategies [72,73]. Additionally, caregiver involvement, simplified medication regimens, and close monitoring may help mitigate the risks associated with frailty, ensuring that elderly patients derive maximum benefit from anticoagulation and other AF therapies [72,73]. By acknowledging frailty as a determinant of clinical decision-making, healthcare providers are better able to align treatment with the unique needs of this vulnerable population.

Finally, a systematic review and meta-analysis [22], involving 285,000 patients, explored how following the recommended care pathway for AF could significantly influence clinical outcomes. The research was a collaborative effort from experts across various institutions, highlighting the importance of adherence to best practices in managing AF for improved patient health [22]. The systematic review and meta-analysis brought critical insights into the clinical outcomes of AF management, particularly concerning adherence to the ABC pathway. First, suboptimal adherence to the ABC pathway was an important finding, with only approximately one in five AF patients receiving care aligned with the recommended pathway [22]. As the previous studies showed, this outcome highlights a significant gap in the practical implementation of guideline-based management strategies. Also, adherence to the ABC pathway was associated with a statistically significant reduction in major adverse outcomes [22]. Specifically, patients following the ABC pathway showed a decreased risk of all-cause mortality, cardiovascular mortality, stroke, and major bleeding [22]. These findings underscore the clinical benefit of a structured, pathway-driven approach to AF management [22]. The study emphasized the importance of comprehensive care for AF patients. Given the frequent multimorbidity and clinical complexity associated with AF, the results advocate for an integrated and holistic management strategy that addresses both the condition and co-existing health concerns. Lastly, the review identified heterogeneity in the definition of the ABC pathway criteria across the included studies [22]. This variability may have influenced the comparability of outcomes and underscores the need for standardized definitions and consistent applications of the pathway in clinical research and practice [22].

More recently, Pearsons et al. (2024) [74] conducted a review that examined the conditions under which integrated care models for AF are most effective, and how these models function in practice. The review identified that integrated care is particularly beneficial for patients at a high risk of complications, such as stroke or heart failure, as well as those requiring continuous management. The study emphasized that the success of integrated care depended on a well-coordinated, multidisciplinary approach, where clear communication among healthcare providers and active patient involvement were key. Additionally, the review highlighted the importance of adapting care strategies to local healthcare contexts and available resources to maximize effectiveness. These findings underscore the need for a flexible, personalized approach to integrated care, tailored to the unique needs of AF patients, to achieve optimal clinical outcomes [43]. Table 1 provides a detailed summary of the studies.

## 3. A Path Forward: Implications for Patient Care and Healthcare Systems

The management of AF has seen significant progress in recent years [43] and proof of this is the AF better care pathway (ABC), the guidelines of the ESC [3]. Building on these recommendations, the 2024 ESC guidelines present the AF-CARE framework [2], which emphasizes Evaluation and Dynamic Reassessment, as a distinct focus area to promote patient-centered care. This development represents a significant evolution from the ABC pathway outlined in the 2020 ESC guidelines [3] for diagnosis and AF management. This approach integrates comorbidity management, stroke prevention, symptom control, and continuous reassessment, allowing for individualized, flexible care plans aligned with patients’ evolving needs [2]. Emphasizing multidisciplinary collaboration, shared decision-making, and education, the ESC guidelines aim to enhance treatment adherence and outcomes [2]. Equity in care is prioritized, addressing disparities and ensuring access to high-quality AF management for all [2]. The ESC updates provide a comprehensive framework to optimize patient care in diverse healthcare settings. So, the future of integrated care in the context of AF is very promising, with the ability to significantly improve patient outcomes and reshape healthcare systems. As the prevalence of AF and other chronic cardiovascular diseases escalates [4,45], integrated care frameworks present a viable and economically sustainable strategy for the long-term management of these conditions [46]. Accordingly, the effective organization of such care requires coordination across primary, secondary, and tertiary care settings, with distinct roles for healthcare personnel at each level [2].

Primary care serves as the foundation for AF management, focusing on early detection, education, and risk factor modification [2,75]. Primary care providers play an essential role in educating patients about AF, empowering them to engage in self-management strategies and lifestyle modifications to reduce AF-related risks [2,75]. Regular monitoring and follow-up in primary care are essential for assessing anticoagulation therapy, managing comorbidities, and ensuring adherence to treatment plans. Furthermore, effective collaboration with specialists is critical for timely referrals, particularly for patients requiring advanced interventions [2,75].

Secondary care settings, such as hospitals and specialized clinics, provide more advanced management for AF patients [2]. This includes optimizing rhythm and rate control. Multidisciplinary teams comprising cardiologists, electrophysiologists, nurses, and pharmacists collaborate to deliver comprehensive care tailored to individual patient needs. Secondary care facilities are also instrumental in risk stratification, particularly in assessing thromboembolism and bleeding risks to guide anticoagulation therapy [2].

Tertiary care is designated for complex cases that necessitate specialized interventions not typically available in primary or secondary settings. This includes advanced procedures like catheter ablation, surgical interventions, and participation in clinical trials for novel therapies [2].

**Figure 1:**
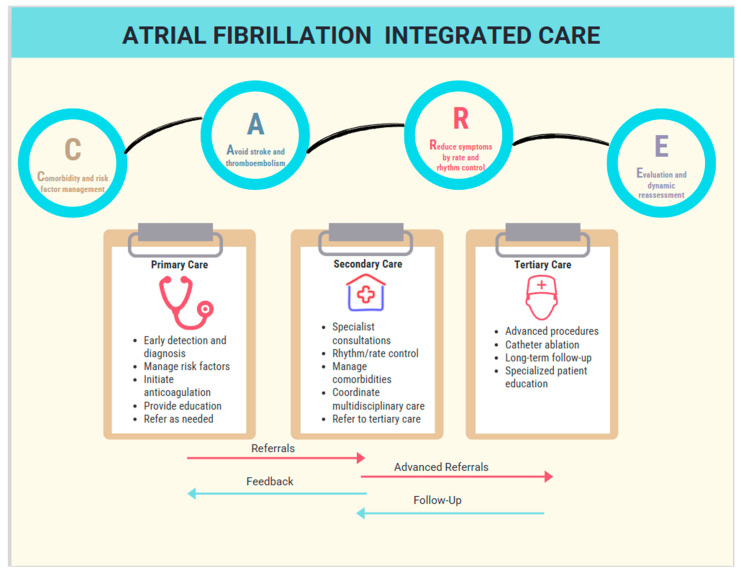
Atrial fibrillation integrated care.

Some healthcare systems may consolidate these into two levels: primary care and specialist care. In such models, specialist care combines the roles of secondary and tertiary care, providing advanced diagnostic and interventions as needed. By organizing integrated care effectively across these levels, healthcare systems can deliver comprehensive and equitable management, ensuring improved outcomes and QoL for patients with AF [2]. The integration process is illustrated in Figure 1.

The atrial fibrillation better care (ABC) pathway has also been reinforced by recent findings from the COOL-AF registry, where Krittayaphong et al. (2024) [76] showed its effectiveness in improving holistic care for patients with AF. This real-world analysis highlights the pathway’s ability to balance stroke prevention, symptom control, and comorbidity management, underscoring its potential to optimize outcomes in diverse patient populations [76]. However, real-world data reveal persistent gaps in anticoagulation management, particularly among frail elderly patients with comorbidities. Bo et al. (2024) [77] identified two critical issues in this population: the underprescription of anticoagulants for patients at risk of thromboembolic events and the frequent use of inappropriately low anticoagulant dosages. These challenges significantly undermine the effectiveness of stroke prevention strategies, particularly in high-risk populations.

The integration of holistic care models, such as those outlined in the ABC pathway [3], may have the potential to address these gaps. By fostering a multidisciplinary approach, involving cardiologists, primary care physicians, specialized nurses, and pharmacists, integrated care models can ensure better adherence to guideline-recommended anticoagulation strategies. Structured follow-ups, patient education, and close monitoring could further enhance the safe and appropriate use of anticoagulants in frail patients. However, the success of these models depends on their consistent implementation, the availability of resources, and the tailoring of care to the specific needs of vulnerable populations. Future studies are needed to evaluate the real-world impact of integrated care in reducing anticoagulation gaps in the most problematic patients and settings.

Patient education is a key to effective AF management, empowering individuals to actively participate in their care and enhancing adherence to treatment strategies. The rise of digital health, such as wearable devices, mobile health technologies (mHealth), and telemedicine has emerged as transformative tools to bridge gaps in patient education and improve the delivery of personalized care [7,55,78]. These technologies provide a practical solution to geographic and logistical challenges and early AF detection ensuring the continuity of care while reducing healthcare costs [79,80]. mHealth applications further extend the reach of telemedicine by offering user-friendly platforms for medication reminders, symptom tracking, and educational content tailored to individual patient needs [57,78]. These tools are particularly beneficial for managing chronic conditions like AF, where long-term adherence to anticoagulation and lifestyle modifications are critical [79]. Of great importance is the realization that the effectiveness of integrated care will depend on its ability to be adapted to each patient. Studies such as Pearsons et al. (2024) [74] have shown that personalized approaches which consider patients’ unique needs and circumstances—such as comorbidities, access to resources, and the health literature—are more likely to succeed. Thus, while mHealth and telemedicine have significant potential, challenges remain, particularly among elderly AF patients who may face difficulties due to limited digital literacy [21]. Bridging this gap requires tailored educational programs, simplified technology interfaces, and support from caregivers to maximize the utility of digital tools. Furthermore, integrating telemedicine and mHealth into multidisciplinary care models ensures patients’benefit from a seamless, coordinated approach to AF management [21].

By incorporating mHealth and telemedicine into patient education strategies, healthcare providers can deliver accessible, personalized, and continuous care, addressing the evolving needs of AF patients in the digital era. These technologies hold the potential to revolutionize patient engagement and improve long-term outcomes when paired with robust support systems and tailored intervention [21].

Future research should continue to explore ways to personalize integrated care to maximize its impact across diverse patient populations. The success of integrated care models relies on effective collaboration between healthcare providers, including primary care physicians, cardiologists, nurses, pharmacists, and other specialists [81,82]. Future efforts should focus on improving communication and coordination among these professionals, ensuring seamless care transitions and the better management of complex, chronic conditions like AF. As the economic burden of chronic diseases like AF grows [20], integrated care offers a cost-effective alternative to the traditional medical model [55]. By reducing hospitalizations, preventing complications, and improving long-term management, integrated care can help alleviate the financial strain on healthcare systems, e.g., Refs. [50,55]. Future research should continue to assess the economic benefits of integrated care, providing evidence for policymakers to implement these models on a wider scale. The adoption of standardized care pathways, such as the ABC pathway for AF, has demonstrated improved outcomes in terms of reduced hospitalizations and better patient QoL [50]. In the future, these models should be expanded and adapted to local healthcare settings, ensuring that all patients with AF benefit from evidence-based management. Additionally, guidelines should continue to evolve based on ongoing research and emerging evidence. Future integrated care models should emphasize patient education and self-management, empowering patients to take an active role in their care. By providing patients with the knowledge and tools to manage their condition, integrated care can enhance patient satisfaction, adherence to treatment plans, and long-term health outcomes [43].

## 4. Conclusions

Integrated care represents a paradigm shift in AF management, transforming it from a traditional, episodic approach to a comprehensive and patient-centered strategy. Future efforts should focus on optimizing these models, addressing implementation challenges, and exploring their applicability across diverse healthcare systems to maximize their impact on the global AF burden. Integrated care for AF showed significant potential in reducing all-cause mortality and cardiovascular hospitalizations compared to usual care [50]. High-risk patients tend to benefit more, suggesting that prioritizing this group could enhance care effectiveness. Despite its promise, the inconsistent use of the term “integrated care” leads to varied interpretations and implementations in clinical practice, affecting outcomes [42]. Additionally, while tools like mobile health technology and nurse-led clinics improve adherence to guidelines and reduce adverse events, these strategies are not uniformly adopted [42]. Models such as the ABC pathway have shown better guideline adherence and improved outcomes [57,61,62,66] and are corroborated by a systematic review and meta-analysis [22], yet their application remains variable. Standardizing integrated care approaches and focusing on high-risk patients, alongside the wider adoption of technology and multidisciplinary teams, is essential for optimizing AF management and improving patient outcomes.

So, the body of evidence underscores the significant potential of structured, multidisciplinary approaches to improving clinical outcomes in this patient population, e.g., Ref. [22]. Across various study designs and settings—from nurse-led interventions to primary care models and mobile health-supported strategies—the findings show consistent benefits, such as reductions in cardiovascular events, hospitalizations, and mortality, e.g., Refs. [50,54]. Despite variations in outcomes between studies, e.g., Refs. [50,51], these results collectively highlight the importance of adherence to clinical guidelines, patient education, and individualized care strategies in achieving superior outcomes compared to usual care. Despite its proven benefits, challenges persist, including suboptimal implementation and regional disparities in care delivery [67,68]. Nevertheless, the evidence supports the integration of multidisciplinary teams and patient education as essential components to improve AF management and overall patient outcomes, e.g., Refs. [22,43,70]. Furthermore, emerging innovations, such as the integration of mHealth technologies [57], offer promising opportunities to enhance the delivery and accessibility of integrated care.

By continuing to innovate, personalize care, incorporate technology, and encourage collaboration between professionals, integrated care models may help meet the challenges posed by AF and other chronic diseases, ultimately contributing to a more sustainable and effective healthcare system.

## Figures and Tables

**Table 1:** Summary of the studies included.

Author(s) and Year	Study Design	Population/Setting	Intervention	Comparison	Key Findings
Inglis et al. (2004) [49]	RCT	152 Patients with chronic AF/Adelaide, Australia	Nurse-led, home-based intervention	Usual post-discharge care	Reduced hospitalizations and mortality rates; long-term improvements in management.
Hendriks et al. (2012) [50]	RCT	712 Patients aged 18+ with AF/Maastricht University Medical Centre	Nurse-led care with decision-support software	Usual care	A 35% risk reduction in cardiovascular events; better guideline adherence.
Wijtvliet et al. (2020) [51]	RCT (RACE 4 trial)	1375 AF patients/multicenter	Nurse-led care with decision-support tools	Standard care by cardiologists	No significant reduction in cardiovascular death or hospital admissions; benefit varied by center experience.
Stewart et al. (2015) [52]	Pragmatic RCT (SAFETY trial)	335 AF patients/multiple centers	Disease-specific intervention with home visits and personalized follow-up	Standard management practices	Reduced hospital readmissions; increased survival rates.
Carter et al. (2016) [53]	Before-and-after study	433 patients with new-onset AF/3 emergency departments in Nova Scotia	Nurse-led AF clinic intervention	Usual emergency department care	Reduced AF-related hospitalizations; improved symptom management and guideline adherence.
Van den Dries et al. (2020) [54]	Cluster RCT (ALL-IN trial)	1240 patients with documented AF/primary care settings in the Netherlands	Integrated care by general practitioners and practice nurses	Usual care by cardiologists	A 45% reduction in all-cause mortality; lower non-cardiovascular mortality.
Guo et al. (2020) [57]	Cluster RCT (mAFA II trial)	3324 AF patients/mobile health (mHealth) technology	Integrated care supported by an AF mobile app	Usual care	Reduced rehospitalization rates and adverse events; enhanced patient outcomes.
Fuenzalida et al. (2017) [58]	Prospective RCT	240 Patients with AF/emergency department in Barcelona	Educational intervention by nurses at discharge	Standard care	Improved patient knowledge; reduced hospital readmissions; better self-management.
Vinereanu et al. (2017) [59]	Cluster RCT (IMPACT-AF trial)	2281 patients across five countries	Quality improvement educational intervention for anticoagulation	Usual care	Increased oral anticoagulant use; reduced stroke incidence.
Bowyer et al. (2017) [60]	RCT	41 AF patients undergoing catheter ablation	Nurse-led structured educational intervention	Standard physician care	Improved QoL and symptom management; reduced anxiety and symptom frequency.
Proietti et al. (2018) [61]	AFFIRM trial post hoc analysis	3169 AF patients (ABC pathway group vs. non-ABC group)	ABC pathway (integrated care)	Non-ABC management	Reduced all-cause mortality, stroke, major bleeding, cardiovascular death, and hospitalizations.
Pastori et al. (2019) [64]	Prospective cohort study	907 AF patients in ATHERO-AF study	ABC pathway (integrated care)	Non-ABC management	Significantly lower rates of cardiovascular events.
Yoon et al., 2019 [65]	Nationwide cohort study	204,842 nonvalvular AF patients in Korea	ABC pathway adherence	Non-ABC management	Improved clinical outcomes; lower all-cause mortality rates.
Proietti et al., 2020 [66]	SPORTIF III and V trial analysis	AF patients with metabolic comorbidities	ABC pathway compliance	Non-ABC management	Reduced cardiovascular events and all-cause mortality in high-risk patients.
Proietti et al., 2021 [62]	ESC-EHRA EORP AF registry analysis	6646 AF patients	ABC pathway adherence	Non-ABC pathway management	Lower risks of cardiovascular events and death.
Luo et al., 2022 [55]	Model-based health economic assessment	Hypothetical cohort of Chinese AF patients	mHealth-based ABC pathway care	Usual care	Cost-effective strategy enhancing ABC pathway implementation.
Koziel et al., 2020 [68]	Real-world study (BALKAN-AF survey)	2712 patients with nonvalvular AF/Balkan region	ABC pathway adherence	Non-ABC care	Improved outcomes but low adherence rates; identified barriers to implementation.
Gumprecht et al. (2020) [69]	Observational registry Gulf SAFE registry	AF patients/Middle East population	Compliance with ABC pathway	Non-compliance with ABC pathway	Compliance with ABC pathway was associated with significant reductions in thromboembolic events, hospitalizations, and mortality rates in the Middle East population.
Bucci et al. (2023) [23]	Prospective registry APHRS-AF registry	4000 AF patients/Asia–Pacific region	Integrated care using the ABC pathway	Usual care or non-compliance with ABC pathway	Compliance with the ABC pathway improved clinical outcomes, including reduced stroke risk, lower mortality rates, and fewer hospitalizations, emphasizing the value of structured care in AF management.
Rivera-Caravaca et al. (2022) [63]	Prospective cohort study Murcia AF project Phase II cohort	AF patients in Phase II cohort/Spain	Implementation of the ABC pathway	Non-compliance with ABC pathway	Adherence to the ABC pathway was associated with improved clinical outcomes, including reduced stroke rates, all-cause mortality, and hospitalizations, underscoring its effectiveness.
Romiti et al. (2021) [22]	Systematic review and meta-analysis	285,000 AF patients	Adherence to the ABC pathway	Nonadherence to the ABC pathway	Nonadherence to the ABC pathway.
Pearsons et al. (2024) [74]	Realist review	Various healthcare settings, AF service delivery	Integrated approach to AF service delivery	Non-integrated or fragmented care	Identified key mechanisms and contextual factors that determine the success of integrated care for AF, emphasizing tailored strategies for specific patient groups and healthcare contexts.

## Data Availability

Not applicable.
